# Supplementary material for: Vitamin D Receptor Gene Polymorphism and Vitamin D Status in Population of Patients with Cardiovascular Disease—A Preliminary Study
Source: Nutrients. 2021 Sep 6;13(9):3117. doi: 10.3390/nu13093117 (PMC8465937; doi:10.3390/nu13093117)
Supplement: Supplementary file 1 [file nutrients-13-03117-s001.zip › nutrients-1342807-supplementary materials.pdf]

Table S1. Primers' sequences, annealing temperature, lengths of products obtained, and results of restriction in PCR reaction

Polymorphism	Starter sequences	Annealing temperature [°C]	PCR product length [bp]	Length of restriction fragments [bp]
**ApaI G/T**	F: 5-CAGAGCATGGACAGGGAGCAA-3<br>R: 5-GCAACTCCTCATGGCTGAGGTCTC-3	60	740	GG: 530 and 210<br>GT: 740, 210 and 530<br>TT: 740
**TaqI T/C**	F: 5-CAGAGCATGGACAGGGAGCAA-3<br>R: 5-GCAACTCCTCATGGCTGAGGTCTC-3		740	CC: 290, 245 and 205<br>CT: 495, 290, 245 and 205<br>TT: 495 and 245
**BsmI G/A**	F: 5-CAACCAAGACTACAAGTACCGCGTCAGTGA-3<br>R: 5-AACCAGCGGGAAGAGGTCAAG GG -3	60	825	GG: 650 and 175<br>GA: 825, 650 and 175<br>AA: 825
**FokI T/C**	F: 5-AGCTGGCCCTGGCACTGACTCTGCTCT- 3<br>R: 5-ATGGAAACACCTTGCTTCTTCTCCCTC-3	60	265	TT: 196 and 69<br>TC:265, 196 and 69<br>CC: 265
